# *Staphylococcus aureus* Regulator Sigma B is Important to Develop Chronic Infections in Hematogenous Murine Osteomyelitis Model

**DOI:** 10.3390/pathogens6030031

**Published:** 2017-07-15

**Authors:** Lorena Tuchscherr, Jennifer Geraci, Bettina Löffler

**Affiliations:** Institute of Medical Microbiology, Jena University Hospital, Jena 07747, Germany; Jennifer.Geraci@med.uni-jena.de (J.G.); bettina.loeffler@med.uni-jena.de (B.L.)

**Keywords:** *sigB*, osteomyelitis, abscess, SCVs

## Abstract

*Staphylococcus aureus* is a major pathogen causing bone infections that can become chronic and difficult to treat. Recently, we described the mechanism employed by *S. aureus* to switch to small colony variants (SCVs) and trigger intracellular bacterial persistence through the global stress regulator SigB. Here, we studied the role of SigB in the formation of chronic osteomyelitis. We used a murine hematogenous osteomyelitis model, where the mice were infected via the tail vein and subsequently developed chronic osteomyelitis. Mice were infected with *S. aureus* LS1, LS1Δ*sigB* and LS1Δ*sigB* complemented and kidney and bone tissues were analyzed six weeks after infection. *S. aureus* LS1Δ*sigB* formed a high rate of abscesses in kidneys, but the bacterial loads and the weight loss of the animals were lower in comparison with animals infected with the wild type and the complemented strain, indicating a more rapid and efficient bacterial clearing by the host immune system. Moreover, the *sigB*-mutant was not able to form SCV phenotypes either in kidney or in bone tissue. Our results demonstrate that staphylococcal SigB is important to avoid bacterial elimination by the host immune response, establish a bone infection and mediate bacterial adaptation (SCV-formation) for persistent infections

## 1. Introduction

*Staphylococcus aureus* is an opportunistic pathogen that is able to trigger a variety of diseases including osteomyelitis, endocarditis and indwelling medical devices. These infections can develop to chronic courses, where they become highly refractory to antibiotic treatment [1,2]. The reason is most likely the complex adaption strategies of *S. aureus* to host tissue. Staphylococcal persistence is associated with a sub-population of phenotypic variants called small colony variants (SCVs) [3]. SCVs grow slowly due to a reduced metabolism and form only very small colonies on agar plates. Recently, we found that SCVs develop in a highly dynamic manner during the infection course and their formation is dependent on the alternative transcription factor sigma B (SigB) [4,5,6]. SigB is known as a stress-induced staphylococcal transcription factor that alters the expression of several genes related with virulence and metabolism. SigB is expressed during the early stationary phase of growth and is involved in the expression of some genes, like adhesins (e.g., *fnbA* induced during the logarithmic phase and *clfA* induced during the transition to stationary phase), but has a negative effect on the expression of genes encoding exoproteins in the late stationary growth (e.g., alpha toxin, hla) [4]. In our recent work, we found that after infection of primary human osteoblasts, the Δ*sigB* mutants were rapidly cleared by the host cells and failed to form SCVs. Moreover, proteomic analysis showed high expression of toxins (such as alpha toxin, hla) by Δ*sigB* [4]. During the pathogenesis of osteomyelitis, many staphylococcal virulence factors define the course of infection [5]. The expression of toxins allows *S. aureus* to establish an acute infection, destroy host tissue, invade deep tissue structures and fight against host immune cells [6,7,8]. Moreover, *S. aureus* develops strategies to hide and persist within host cells/tissue to establish a chronic infection. In this study, we analyzed the role of SigB during an acute hematogenous infection that develops to a chronic osteomyelitis in mice. We demonstrate that the lack of *sigB* induced high numbers of abscesses which contributed to the clearance of the bacteria. Consequently, *SigB* represents a potential target to eliminate *S. aureus* and prevent the development of SCVs associated with chronic osteomyelitis.

## 2. Materials and Methods

### 2.1. Strains for Infection Models

*S. aureus* wild-type LS1, Δ*sigB* and the complemented *sigB* strain were used in our study. The characterization of these strains was described recently [4]. For the infection experiments, overnight cultures of the *S. aureus* strains LS1, Δ*sigB* and the complemented *sigB* (Δ*sigB* complemented) were prepared in 30 mL of brain heart infusion (BHI) broth and incubated at 37 °C and 160 rpm (for each strain). After two washing steps with PBS and centrifugation at 5000 rpm, the concentration of each strain was adjusted to an OD of 1 at 578 nm. Cell pellets were resuspended in PBS. To control the concentration of the inoculum, the number of CFU/mL was determined by plating in 10-fold serial dilutions on blood agar. All strains presented only wild type and not SCV phenotype at the time of administration.

### 2.2. Haematogenous S. aureus Osteomyelitis Model in Mice

Groups of 6-10 C57bl-6 mice 10 weeks old female were infected with *S. aureus* LS1, LS1Δ*sigB* and the complemented mutant for *sigB* (Δ*sigB* complemented), which have been described and characterized previously [4]. The model has already been described by Horst et al. [9]. Briefly, the mice were infected intravenously with 10^6^ CFUs of *S. aureus* in 150 μL of PBS. Mice were sacrificed after 6 weeks post infection by CO_2_ asphyxiation at the indicated time of infection. The animals were maintained in individually ventilated cages and were given food and water *ad libitum*. All experiments were approved by the North Rhine-Westphalia Agency for Nature, Environment, and Consumer Protection (Landesamt für Natur, Umwelt und Verbraucherschutz Nordrhein-Westfalen-LANUV; permit number 84-02.04.2012.A293). For enumeration of bacteria in bones (femur and tibia) and kidneys of infected mice were transferred to one tube with 2 mL PBS and homogenized mechanically on ice (polytron pt2500, Fisher Scientific, Schwerte, Germany). The homogenized tissues were plated in 10-fold serial dilutions on blood agar and the plates were incubated at 37 °C. The percentages of big WT and SCV-like colonies of the intracellular surviving bacteria were determined by a colony counter (Biocount 5000, BioSys GmbH, Karben, Germany). All colonies with a diameter <0.6 mm were considered as SCVs (<5 and <10-fold smaller than those of the wild-type phenotypes, respectively). Due to the slow formation of SCVs, the final values of the amount of SCVs on agar were determined after 72 h of incubation. The animals were weighted daily during the first 22 days. The weight changes of infected mice were assessed by measuring the mice pre-infection and 7 days post-infection because after this point the animals died or recovered the weight.

### 2.3. Statistical Analysis

Data were analyzed using GraphPad Prism software version 5 (GraphPad Software, La Jolla, CA, USA). Data are expressed as means ± SD. Comparison between groups was performed by use of a one-way analysis of variance test followed by Turkey’s multiple comparisons test. The *p* value was interpreted as: ns *p* > 0.05; * *p* > 0.05; ** *p* > 0.01; *** *p* > 0.001 and **** *p* > 0.0001.

## 3. Results and Discussion

### 3.1. SigB Is Required for S. aureus Persistence in Bone and Kidney Tissue

**Figure 1:**
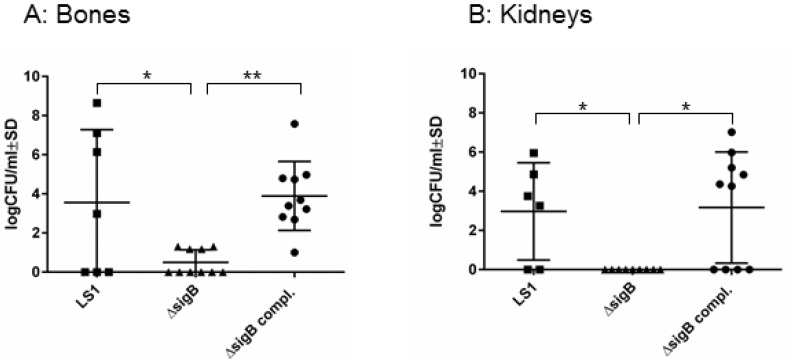
*SigB* is necessary for persistence of *S. aureus* in bone and kidney tissues. Bacterial persistence of LS1, Δ*sigB* and Δ*sigB* complemented was analyzed in a murine chronic osteomyelitis model 6 weeks p.i. Bones (**A**) and kidneys (**B**) were homogenized and plated on agar plates for counting the CFUs on the following day. The results represent the means ± SD and were analyzed by ANOVA test and Turkey’s as multicomparison test.

**Figure 2:**
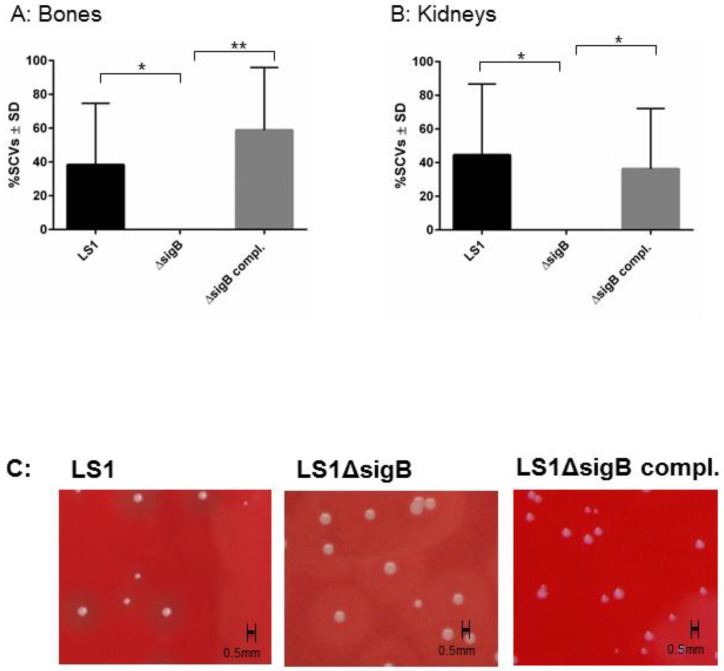
The Δ*sigB* mutant does not form SCV phenotypes. The phenotypic diversity was analyzed on blood agar plate after 48 and 72 h of incubation. The percentage of small and very small (SCV) phenotypes (all colonies with a diameter <0.6 mm with size <5 and <10-fold smaller than those of the wild-type phenotypes, respectively) recovered (between 200 and 500 colonies examined in each sample) were determined after 6 weeks p.i. from homogenized bones (**A**) and kidneys (**B**) infected with LS1, Δ*sigB* and Δ*sigB* complemented strains. The values represent the means ± SD and were analyzed by ANOVA and Turkey’s test for multiple comparison. **C**) Photographs of recovered colonies were performed after infection of C57/Bl6 mice with strains LS1, LS1∆*sigB* or LS1∆*sigB* compl.

To study the function of SigB in the course from acute to chronic infection, we performed a hematogenous murine model of chronic *S. aureus* infection. After 6 weeks post infection, we recovered disseminated bacteria from kidneys and bones and the enumeration of CFU/mL was analyzed on blood agar (Figure 1). In both tissues LS1 persisted in significant numbers after 6 weeks. By contrast, LS1Δ*sigB* was almost cleared whereas this effect could be fully reversed by the complementation of *sigB* (Figure 1A,B, Table 1). Furthermore, in both tissues a high percentage of SCVs was found indicating *S. aureus* adaptation (Figure 2A–C). However, after infection with LS1Δ*sigB* the formation of SCVs was completely absent. These results are in line with previously published cell culture models [4,10] (Figure 2C). The recovery of CFUs was higher in bone than in kidneys, indicating a preference of *S. aureus* for bone tissue during long-term persistence (Figure 1A,B). Furthermore, the SCV formation was observed in both tissues showing the ability of *S. aureus* to adapt to different types of host cells/tissue (Figure 2A,B). By using our rat local osteomyelitis model, where *S. aureus* was directly inoculated in bone[4], we demonstrated that the mutants were not able to establish a local bone infection. In our hematogenous murine model we could analyze the role of sigB during the switch from acute to chronic infection. Taken together, our results demonstrate a crucial role of SigB in establishing a chronic infection, particularly in bone tissue.

### 3.2. Mutation of sigB Induced Several Abscess in Kidneys and Enhanced Bacterial Clearing

**Figure 3:**
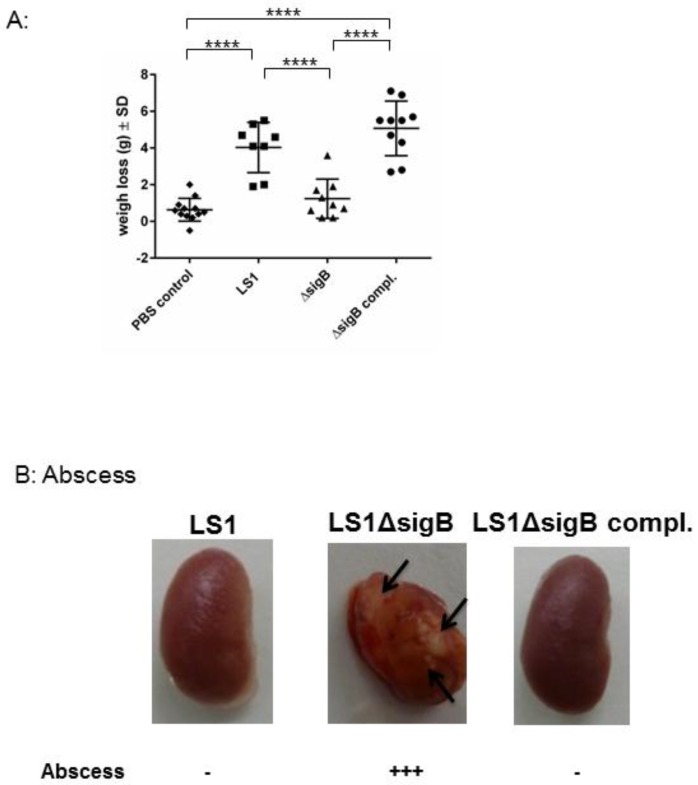
The absence of *sigB* in *S. aureus* induced abscess formation in kidneys and early recovery of the infected animals. Mice were infected via tail vein with LS1, Δ*sigB* and Δ*sigB* complemented strains. (**A**) The body weights of surviving mice were monitored for 21 days and recorded at each time point. Here we represent the difference between the day 0 immediately after infection and 7 days post infection. The PBS group showed almost no change in weight. In contrast, mice in the LS1 and Δ*sigB* complemented strains group showed a significant decrease in body weight during the 7-day period in comparison with PBS and Δ*sigB* groups (*p* < 0.05). There was no significant difference in body weight between the Δ*sigB* and PBS groups (*p* > 0.05). One-way analyses of variance (ANOVA) followed by the Tukey test were used to compare multiple groups. (**B**) The photographs of recovered kidney after 6 weeks post infection are shown. The arrows indicate the localization of abscess. The amount of abscess was estimated by eye observation.

To understand the role of *sigB* during the whole course of infection, we analyzed the host response, such as abscess formation and the weight changes of all infected animals. *S. aureus* WT induced a systemic infection reflected by a high weight loss (Figure 3). By contrast, the mice infected with *S. aureus* Δ*sigB* showed only a moderate weight change in comparison with mice infected with the WT strain. *S. aureus* Δ*sigB* complemented induced a similar effect as the WT strain LS1. Moreover, the kidneys from mice infected with Δ*sigB* mutant showed several abscesses after 6 weeks compared with mice infected with WT or complemented Δ*sigB* strain. It is well known that the absence of *sigB* has an enhancing effect on *agr* that results in increased expression of proinflammatory virulence factors [11]. Bischoff et al*.* described that *sigB* reduces *agr* expression in a growth phase-dependent manner [11]. Thus, the absence of *sigB* induces upregulation of staphylococcal toxins and downregulation of adhesins [10,11,12]. Consequently, LS1Δ*sigB* can cause a high inflammatory response resulting in massive abscess formation and in complete elimination of bacteria directly in kidneys [5,7,11]. Abscess formation is the result of immune host cells recruited by staphylococcal virulence factors [13]. Consequently, the high virulence expression due to the mutation of *sigB* enhances the elimination of *S. aureus* during the blood passage in kidneys which is reflected by the formation of abscess. Accordingly, *sigB* enhances the formation of abscess that apparently promotes the elimination of *S. aureus*.

## 4. Conclusions

The staphylococcal global stress regulator SigB is a central factor for establishing chronic staphylococcal bone infections. The lack of SigB induces a high expression of secreted virulence factors[5], which contribute to abscess formation where most of the bacteria are efficiently eliminated. *S. aureus* is a multifactorial pathogen and it has different strategies to survive in the host. The therapy designed to target one virulence factor will fail to eliminate or treat staphylococcal infections. Recently, it was described that *sigB* mutants were more susceptible to different antimicrobials [14,15] ]. A possible combination between a vaccine where SigB is included as target in combination with an antimicrobial treatment might be a good alternative for treating chronic staphylococcal infections. Consequently, SigB is a potential target for novel antimicrobial strategies against invasive and persisting infections. However, further investigation is necessary in this field.

## Figures and Tables

**Table 1:** Survival rate for each strain. The survival rate was calculated in percentage taking in consideration that the initial bacterial inoculum was in log = 6.67 CFU/mL (this amount was taken as 100%).

Strain	Bone	Kidney
**LS1**	54.3%	43.6%
**LS1Δ*sigB***	7.26%	0%
**LS1Δ*sigB* complemented**	55.7%	46.4%
